# Treating Cattle to Protect People? Impact of Footbath Insecticide Treatment on Tsetse Density in Chad

**DOI:** 10.1371/journal.pone.0067580

**Published:** 2013-06-14

**Authors:** Noël Ndeledje, Jérémy Bouyer, Frédéric Stachurski, Patrice Grimaud, Adrien Marie Gaston Belem, Fidèle Molélé Mbaïndingatoloum, Zakaria Bengaly, Idriss Oumar Alfaroukh, Guiliano Cecchi, Renaud Lancelot

**Affiliations:** 1 Institut Universitaire des Sciences et Techniques d'Abéché (IUSTA), Abéché, Tchad; 2 Centre International de Recherche-Développement sur l’Elevage en Zone subhumide (CIRDES), Bobo-Dioulasso, Burkina Faso; 3 Laboratoire National d’Elevage et de Recherches Vétérinaires, Institut Sénégalais de Recherches Agricoles, Dakar, Sénégal; 4 Unité Mixte de Recherche Contrôle des Maladies Animales Exotiques et Emergentes, Centre de Coopération Internationale en Recherche Agronomique pour le Développement (CIRAD), Montpellier, France; 5 Unité Mixte de Recherche 1309 Contrôle des Maladies Animales Exotiques et Emergentes, Institut National de la Recherche Agronomique (INRA), Montpellier, France; 6 Institut de Recherche en Elevage pour le Développement (IRED), N’Djamena, Tchad; 7 Université Polytechnique de Bobo-Dioulasso (UPB), Bobo-Dioulasso, Burkina Faso; 8 Food and Agriculture Organization of the United Nations, Animal Production and Health Division, Rome, Italy; Instituto de Higiene e Medicina Tropical, Portugal

## Abstract

**Background:**

In Chad, several species of tsetse flies (Genus: *Glossina* ) transmit African animal trypanosomoses (AAT), which represents a major obstacle to cattle rearing, and sleeping sickness, which impacts public health. After the failure of past interventions to eradicate tsetse, the government of Chad is now looking for other approaches that integrate cost-effective intervention techniques, which can be applied by the stake holders to control tsetse-transmitted trypanosomoses in a sustainable manner. The present study thus attempted to assess the efficacy of restricted application of insecticides to cattle leg extremities using footbaths for controlling *Glossina m. submorsitans,* *G* *. tachinoides* and *G* . *f* *. fuscipes* in southern Chad.

**Methodology/Principal Findings:**

Two sites were included, one close to the historical human African trypanosomiasis (HAT) focus of Moundou and the other to the active foci of Bodo and Moissala. At both sites, a treated and an untreated herd were compared. In the treatment sites, cattle were treated on a regular basis using a formulation of deltamethrin 0.005% (67 to 98 cattle were treated in one of the sites and 88 to 102 in the other one). For each herd, tsetse densities were monthly monitored using 7 biconical traps set along the river and beside the cattle pen from February to December 2009. The impact of footbath treatment on tsetse populations was strong (*p* < 10^-3^) with a reduction of 80% in total tsetse catches by the end of the 6-month footbath treatment.

**Conclusions/Significance:**

The impact of footbath treatment as a vector control tool within an integrated strategy to manage AAT and HAT is discussed in the framework of the “One Health” concept. Like other techniques based on the treatment of cattle, this technology should be used under controlled conditions, in order to avoid the development of insecticide and acaricide resistance in tsetse and tick populations, respectively.

## Introduction

In Chad, agriculture accounts for over 50% of the Gross Domestic Product (GDP), corresponding to 10 billion US$ [1], and provides employment to 65% of the economically active population [2]. Livestock plays a major role in the agriculture of Chad, with an estimated standing stock of approximately 18 million ruminants: 7.2 million cattle, 1.4 million camels, 6.5 million goats, and 3 million sheep [2]. Livestock generates income for 40% of the rural population, corresponding to 18% of GDP [3].

African animal trypanosomoses (AAT), a group of parasitic diseases transmitted by tsetse flies (Genus: *Glossina* ), represents one of the major obstacles to the development of more productive farming systems. Three species of tsetse are known to be present in the country, namely *Glossina* *morsitans* *submorsitans,* *G* *. tachinoides* and *G* *. fuscipes* *fuscipes* [4,5]. Although recent, large-scale field data on tsetse distribution are lacking [6], entomological prediction maps [7] and recent unpublished updates of global livestock distribution datasets [8] indicate that an area of 65,000 km^2^ may be infested by tsetse flies and that one million cattle may be at risk of tsetse-transmitted trypanosomosis.

In addition to AAT, human African trypanosomosis (HAT), or sleeping sickness, also continues to impact public health in Chad, with 2,980 cases reported from the years 2000 to 2009 [9]. HAT cases in Chad are caused by *Trypanosoma bruceigambiense* and they are reported from the southern foci of Bodo and Moissala, associated to the rivers Mandoul and Nana Barya [9]. Overall, 465,000 people distributed over an area of 14,000 km^2^ are estimated to be at risk of contracting HAT [10]. In 1990, the Chadian government launched a national programme to eradicate sleeping sickness; the programme included information campaigns, education and interventions against the vector. However, as it was the case for AAT, HAT control in Chad was largely based on curative drugs.

Past campaigns against tsetse, based on bush clearing, game destruction, and residual spraying of insecticides (dichlorodiphenyltrichloroethane — DDT-dieldrin and pyrethroids) [11,12] failed to eradicate the vectors in Chad. Presently, the government is focusing on more sustainable and cost-effective intervention techniques, such as insecticide treated targets and live baits [13], which can be applied by the stake holders. Applied research efforts are underway and in particular a collaboration has been initiated between the Institut de Recherche en Elevage pour le Développement (IRED) (ex *Laboratoire de Recherches Zootechniques et Vétérinaires de Farcha*) in N’djamena, Chad and the *Centre International de Recherche-Développement sur l’Elevage en zone Sub-humide* (CIRDES) in Burkina Faso, to develop innovative control methods against tsetse flies.

Restricted application of insecticide to the preferred tsetse feeding areas on animal bodies, i.e. leg extremities [14,15], has been suggested to limit the cost of treatment while preserving the demonstrated efficacy of the live bait technique [16]. Indeed, restricted application of insecticide has been recognized as a cheap, safe, and environmentally-friendly farmer-based method to control tsetse and animal trypanosomoses [17]. It has also been suggested that it might be efficient to control Rhodesian sleeping sickness, caused by *T* . *b* *. rhodesiense* , for which cattle can represent an important reservoir [18]. In addition to partial spraying, the footbath is another technology that could be adapted to treat the legs of cattle with pesticides. Footbath restricted treatment was initially developed to control the hard tick *Amblyomma* *variegatum* [19]. It must be noted that, as for any control method using acaricide, an inappropriate use could lead to acaricide resistance in tick populations [20]. The risk is, however, limited against *A* *. variegatum* because of its invasion behaviour, leading to prolonged contact with high doses of acaricide (see below).

Feeding by tsetse concentrates on the legs of cattle particularly the forelegs [14]. Previous experiments documented that restricted application of alpha-cypermethrin at 0.005% using footbaths or by partial spraying of the lower parts of the animal body was as efficient and as persistent as full body spraying to kill *G* . *p* *. gambiensis* and *G* *. tachinoides* [14], and efficient enough to control *G* . *p* *. gambiensis* , *G* *. tachinoides* and *G. m. submorsitans* [21]. Under field conditions in Burkina Faso, restricted application of deltamethrin 0.005% using footbaths allowed a rapid decrease in the apparent densities of *G* . *p* *. gambiensis* and *G* *. tachinoides* [14]. Other field tests in Burkina Faso showed that restricted application of deltamethrin 0.005% using footbaths led to a significant difference in the annual incidence of AAT between a control herd (76%) and a treatment herd (11%) [16]. Following on these results, almost 40 footbaths were built in Burkina Faso by development projects to control AAT [22].

The footbath technique has never been tested in central Africa, particularly against *G. m. submorsitans* and, even more importantly, against *G* . *f* *. fuscipes* ; the latter is considered the most important vector of sleeping sickness in central Africa. The specific objective of the present study was to assess the field efficiency of footbaths to control *G. m. submorsitans* *,* *G* *. tachinoides* and *G* . *f* *. fuscipes* in southern Chad. Two sites were included, one close to the historical HAT focus of Moundou and the other in the proximity of the active HAT foci of Bodo and Moissala. The overall objective was to assess if the footbath technique might offer an opportunity to control both AAT in cattle and HAT in humans, following the One Health concept.

## Materials and Methods

### Ethical statement

Sample collection was approved by the Comité de pilotage, de suivi et d’éthique of the National Agricultural System of Chad, which reviews and approves all field and experimental vet protocols carried out by IUSTA (Institut Universitaire des Sciences et Techniques d’Abéché). Cattle belonged to local farmers, and insecticide treatment was carried out with the cattle owners’ permission.

Tsetse flies were collected in traps laid along the river system on national territory, where IUSTA has a mandate to keep track of entomological tsetse densities.

### Study sites

**Figure 1:**
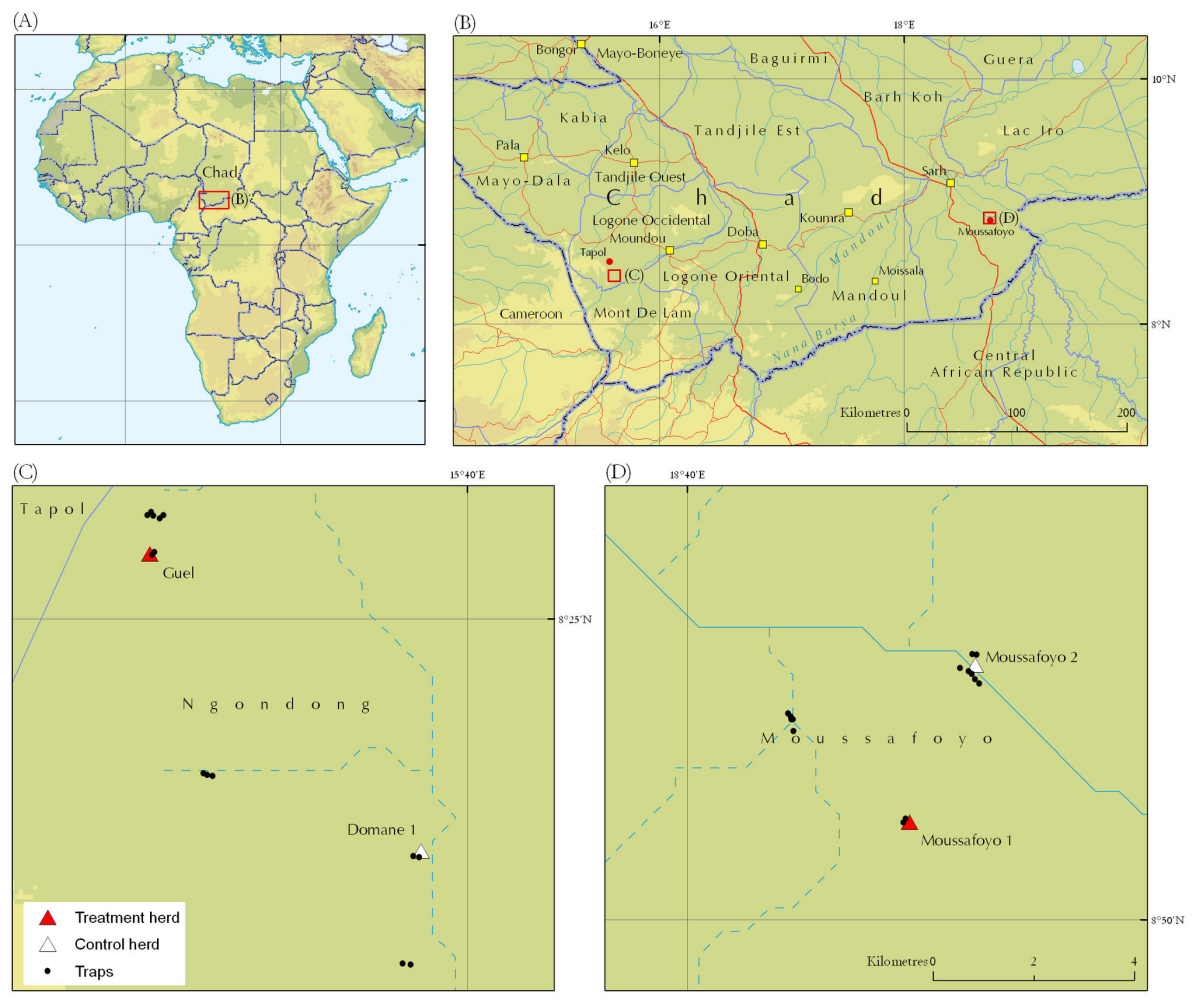
Study sites for impact assessment of footbath insecticide treatment on tsetse apparent density in Chad. The red boxes in panels A and B show the global and specific study areas, respectively, corresponding to panels C and D. The hydrological network shown in sections B, C and D is the HydroSHEDS 15-arcsecond river network of Africa [49].

The maps depicted in Figures 1A and 1 B show the general location of the study areas in Africa and Chad, respectively. From October to December 2008, regular missions allowed to identify herds close enough to be submitted to similar tsetse challenge, but spatially independent (absence of common herd water points and grazing area). The first site (Figure 1C) was located in the vicinity of Tapol, region of Moundou. At this site, the footbath-treated herd was located in the village of Guel, and the negative control herd was in the village of Domane 1. The second site (Figure 1D) was located in the vicinity of Moussafoyo, in the region of Sarh, close to the border with the Central African Republic (CAR). The treated herd was located at the site designated Moussafoyo 1 and the negative control herd was at the site designated Moussafoyo 2.

At both sites mean annual rainfall was 1,200 mm, with a rainy season stretching from May to September. Most of the farmers in these villages were settled and their cattle were mainly used for draught power. At each site, the treatment and control herds were located 4 to 8 km apart and they were watered at different sections of the same river. Their grazing areas were also distinct.

In Moundou, the riverine vegetation along the tributaries serving as water points is a closed gallery forest whereas in Sarh, the river is much bigger and the riverine vegetation is open. At both sites, the neighbouring vegetation is constituted by dry forests, woody and bushy savannah, and strong human encroachment existed. Tsetse habitats are very much fragmented by crops. During the rainy season, tall grasses grow in the savannah areas allowing tsetse to disperse around the river course due to an increase of the relative humidity.

### Entomological monitoring

At each site, 7 biconical traps [23] were set along the river and beside the cattle pen from February to December 2009 (Figure 1 C& D). In Guel (treatment site), five traps were set every 100m along one single river section located at ~1km from the cattle pen and two others at 40 and 60m from the cattle pen. In Domane 1 (negative control), two river sections were monitored; the first with three traps set every 100m and located at 4.4km from the cattle pen and the second with two traps set at 180m from each other and located at 2.2km from the cattle pen. Two traps were set at 130 and 180m from the cattle pen. In Moussafoyo 1 (treatment site), five traps were set along a single 360m river section located at 3km from the cattle pen, at distances between 50 and 200m from each other. Two other traps were set at 100m from the cattle pen. In Moussafoyo 2 (negative control site), one single river section of 500m located at ~300m from the cattle pen was monitored using 5 traps set every 80 to 140m. Two traps were set at 200m from the cattle pen.

Tsetse flies were collected every 48 h during 6 days, at a monthly frequency. After capture, flies were identified to the species level.

### Initial serological prevalence

In order to evaluate the trypanosomoses risk, 50 animals were selected randomly from each herd in November 2008 to obtain blood samples. Samples were tested for *Trypanosoma bruceibrucei*, *T. congolense* and *T* *. vivax* with the indirect ELISA-method that detects anti-trypanosome antibodies in plasma, to ascertain past or present infections with trypanosomes [24].

### Cattle treatments

**Figure 2:**
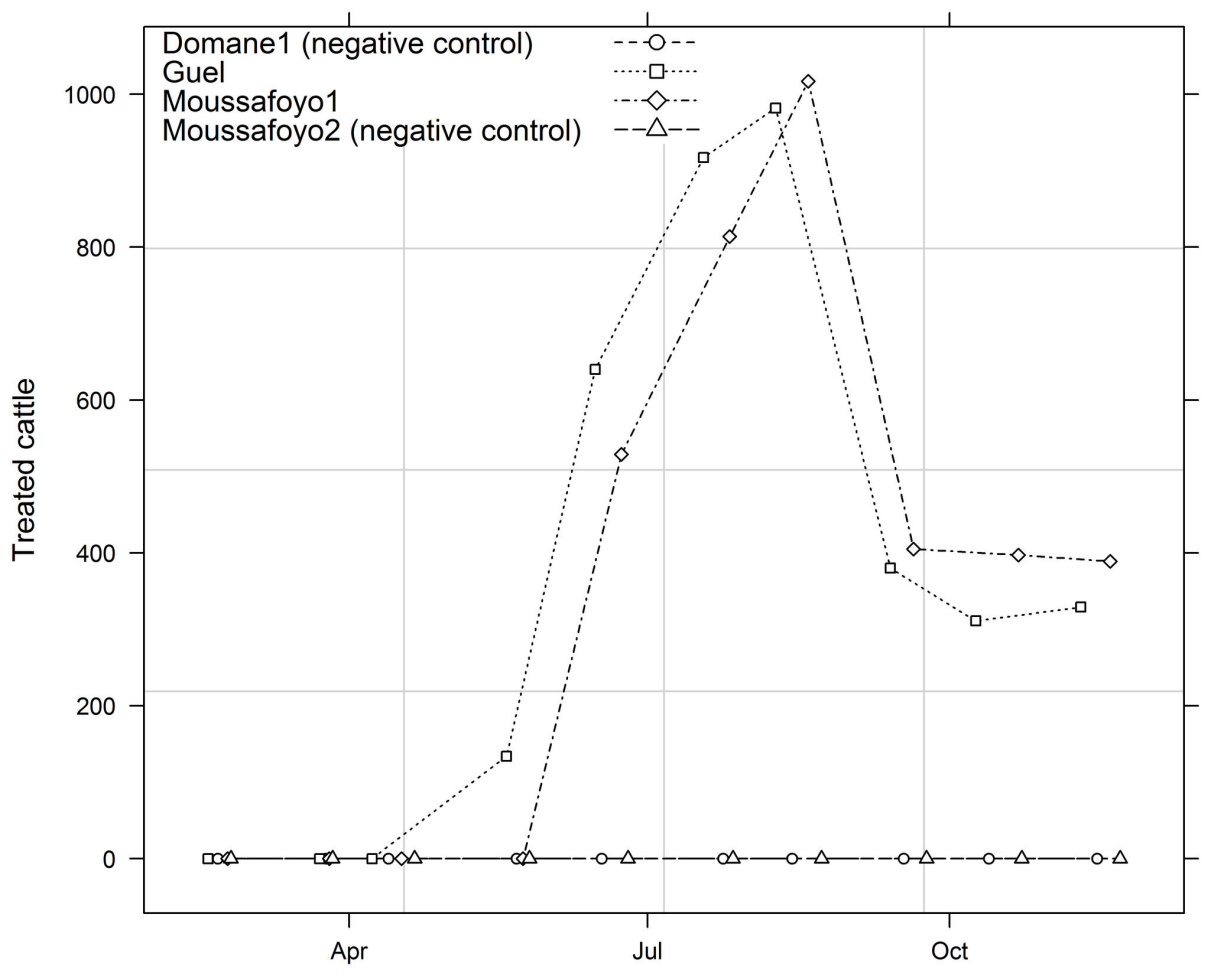
Monthly number of treated cattle at the four study sites for the impact assessment of footbath insecticide treatment on tsetse apparent density in Chad. Points were drawn at the dates corresponding to the visits.

Animals were treated against AAT following a footbath protocol described previously in the study of Stachurski & Lancelot [19] using a formulation of deltamethrin 0.005% (1 mL/L water, Vectocid^ND^) according to label instructions from 28 May 2009 at Guel and 12 June 2009 at Moussafoyo 1. Footbaths were ~3.5m long, 1m wide, and 0.4m deep. The level of the formulation was adjusted at ~19cm high before each treatment, which corresponded to a volume of ~240L. The mean uptake by treatment per head of cattle was estimated at 200 mL. Animals were treated every 3 days from June to August, and weekly from September to December. The number of animals by treatment varied from 67 to 98 in Guel and 88 to 102 in Moussafoyo 1. The total number of animals treated per month at each site is presented in Figure 2. A total of 3,693 and 3,551 individual treatments were recorded in Guel and Moussafoyo1, respectively.

Treatment time was not recorded during this study. In similar conditions in Burkina Faso however, 60 cattle were treated in ~8 min [14].

### Statistical analysis

A Poisson distribution was used for modelling the response variable which was the total *Glossina* catches for 6 days. Observations were repeated for each trap, thus introducing a between-observation dependency. To account for this, we used a Poisson regression model with random effects [25]. The only random effect was the trap, representing the deviation of trap *j* on the mean (unknown) tsetse apparent density *d *_*i j*_ for a given capture event *i*:

log(*d *_*i j*_) = *b*_0, j_ + *b*_1_ *x*_1, i j_ + … + *b *_*p*_ *x *_*p, i j*_

Model intercept was *b*_0; j_ = *b*_0_ + *u*_*j*_ where *b*_0_ was the overall population mean, and *u *_*j*_ was the random effect associated with trap *j*. It was assumed that *u *_*j*_ was distributed according to a Gaussian distribution with a null mean and a variance υ^2^ (to be estimated). The numbers *b*_0_, *b*_1_ … *b*_p_ were the fixed-effect coefficients (to be estimated). The full model was *y *_*i j*_ = *d *_*i j*_ + *e *_*i j*_, where *y *_*i j*_ was the observed count, assumed to follow a Poisson distribution conditionally on trap *j* and explanatory variables; *e *_*i j*_ was the residual error.

The explanatory variables were the time (in months) from the beginning of the treatment, the treatment type (negative control i.e. without any insecticide treatment or footbath), and the region (Sarh or Moundou).

Data analysis was performed with the R software [26] and the additional package lme4 [27].

## Results

### Tsetse species

The total number of tsetse flies captured during experiments was 921. The species composition was different between sites. In Moundou, the two species captured were *G* . *f* *. fuscipes* (390 flies) and *G* *. tachinoides* (42), corresponding respectively to 90.3% (s.d. 1.4%) and 9.7% (s.d. 1.4%) of the captures. In Sarh, three species, i.e. *G* . *f* *. fuscipes* (223), *G. m. submorsitans* (136) and *G* *. tachinoides* (130) were captured corresponding to 47.5% (s.d. 2.3%), 29.0 (s.d. 2.1%) and 27.7% (s.d. 2.1%) of the captures, respectively.

### Serological prevalence

For *T* *. vivax* , the serological prevalence was not different between Moundou and Sarr (χ^2^ = 3.4, df = 1, p-value = 0.07), and it averaged 14% (s.d. 2%). The mean prevalence in Moundou was 9% (s.d. 3%) and no difference was observed between treatment and negative control site (χ^2^ = 0.49, df = 1, p-value = 0.48), with a mean prevalence of 9% (s.d. 3%). In Sarh however, the prevalence was higher (χ^2^ = 12.7, df = 1, *p* < 10^-3^) in Moussafoyo 1 (treated herd, 34%, s.d. 7%) than in Moussafoyo 2 (negative control, 4%, s.d. 3%).

For *T. congolense*, the serological prevalence was nil in all sites whereas only one animal was positive for *T. brucei* s.l. in Moussafoyo 1 (2%, s.d. 2%).

### Tsetse apparent densities before treatment

**Figure 3:**
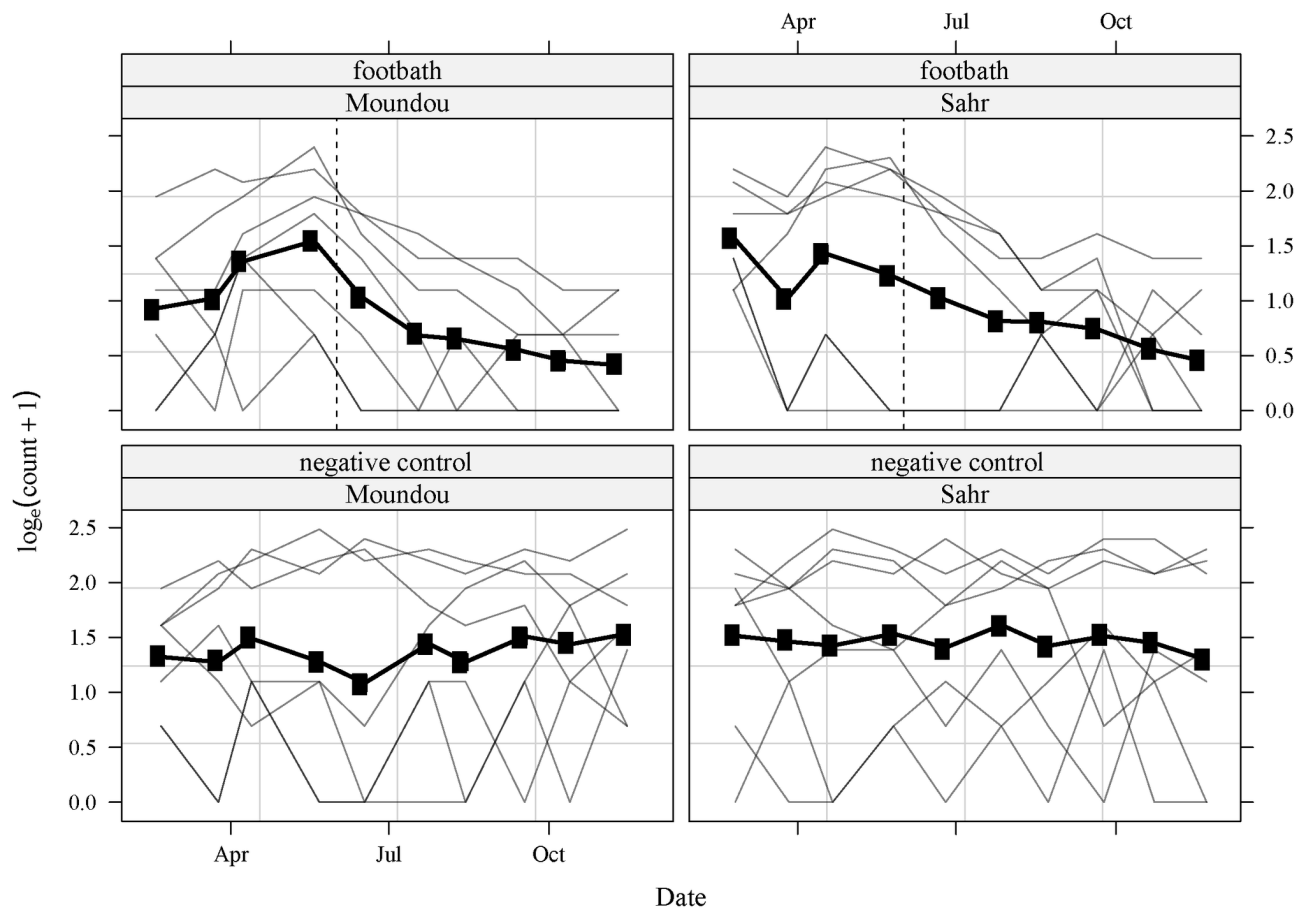
Tsetse total catches (**log scale**) at the treatment (**top**) and control (**bottom**) sites in the two regions of southern Chad. The black line represents the mean observed values; the light grey lines depict the observed values for the individual traps, and the black vertical dashed line indicates the start of footbath treatments.

Prior to experimentation, the total catches were similar between Moundou and Sarr (*p* = 0.64), and between negative control and footbath treatments (*p* = 0.93). Temporal variations were not significant at the site level (*p* = 0.24, Figure 3), and the mean apparent density (flies per trap and per day) was 0.64 (s.d. 0.14). However, at the trap level, there were important temporal variations; tsetse densities increased and decreased in the traps located far from and close to the river, respectively, during the rainy season.

### Impact of footbath treatments

The “region” (Moundou and Sarr) fixed effect was not significant (*p* = 0.62) and this variable was removed from the model, which included time, treatment type, and interaction between these variables as the fixed effects (Table 1), and trap number as the random effect.

**Table 1:** Fixed-effects coefficients of a mixed-effect Poisson model of the impact of footbath treatment of cattle on total catches of tsetse in Chad.

Fixed effects		Estimate	Std. Error	z value	Pr(>|z|)
Intercept		1.206	0.256	4.709	2.5 10^-6^
Time (in months)		0.006	0.031	0.201	0.841
Footbath treatment (ref. categ. = control)		-0.564	0.387	-1.458	0.145
Interaction between time and footbath treatment		-0.262	0.066	-3.982	6.8 10 ^-5^

The impact of footbath treatment on tsetse total catches was strong and significant (Table 2, interaction time * treatment footbath, *p* < 10^-3^). Table 2 presents the monthly reduction of tsetse total catches using the catches of the former month as baseline. Overall, footbath treatment resulted in a 80% reduction of tsetse total catches within 6 months.

**Table 2:** Predicted changes in total catches of tsetse in Chad following cattle footbath treatment, using a mixed-effect Poisson model.

Time after treatment (months)	Total tsetse catches (number)	Monthly reduction in tsetse total catches (number)
0	1.9	
1	1.5	-0.4
2	1.1	-0.3
3	0.9	-0.3
4	0.7	-0.2
5	0.5	-0.2
6	0.4	-0.1

Fitness of the predictions by the model was adequate in general (Fig. Sl, panel a), but slightly overestimated for small values and underestimated for high values. In addition, random effects were slightly asymmetrical in comparison to a normal distribution (Figure S1, panel b). Some traps performed differently from others (Figure S1, panel c), probably as a consequence of the patchy pattern of tsetse fly distribution. However, the impact on the estimation of the fixed-effect coefficient was limited so all the data were kept in the analysis.

## Discussion

Footbath treatment of cattle using deltamethrin 0.005% allowed a strong and significant reduction of tsetse apparent densities in Chad at the two study sites where *G* . *f* *. fuscipes* and *G. m. submorsitans* are the predominant species. Even though the preferred feeding sites of *G* . *f* *. fuscipes* have not been studied, it is likely that the legs are a predilection feeding site for most tsetse species because that location provides the flies a means to evade defence behaviors of cattle. Actually, tsetse are very easily disturbed during feeding as they cannot afford high mortality rates during blood intake [28].

The study sites are close to a historical and still active sleeping sickness foci where *G* . *f* *. fuscipes* is the main disease vector [29]. Results are in agreement with those obtained using footbath treatments against *G* . *p* *. gambiensis* , *G* *. tachinoides* and *G. m. submorsitans* in Burkina Faso [14,16,30], and in Zimbabwe using partial spraying against *G* *. pallidipes* and *G* . *m* *. morsitans* [15]. This confirms that treating cattle using restricted application of insecticide may allow an overall reduction in tsetse density and thus trypanosome challenge. The impact of footbath treatment was monitored during 6 months which means that the treatment repeatedly reduced tsetse densities every month, as shown by the absence of significance of the "Time" fixed effect. This study design is classical in field control studies [14,16]. Tsetse have a k-demographic strategy and their densities are thus not subjected to important seasonal or annual changes [31]. It is thus unlikely that the same experiment conducted another year would give different results. The apparent tsetse densities were recorded using biconical traps, which are not as efficacious for the different tsetse species trapped in this study as they are for catching *G* . *f* *. fuscipes* and *G* *. tachinoides* (efficacy around 0.01/sq. km. /day for the latter species) [32]. Moreover, the percentage of tsetse attracted to the trap that actually enter it is generally low (<0.2) [33], and tsetse challenge for cattle is thus probably underestimated. Moreover, tsetse trapping rates are not independent of fly population densities, which may prevent an accurate estimation of very low population densities.

Considering tsetse apparent densities and serological prevalence of trypanosomes at the study sites, the risk for trypanosomoses can be considered low to medium [34,35]. Wild hosts were very scarce, tsetse densities relatively low, and their populationsfragmented, which corresponds with a typical endemic cycle for AAT [36]. Moreover, landscape fragmentation reduces the speed of re-invasion of the cleared water points along rivers by reducing tsetse dispersal capacities [37]. All these factors provide ideal conditions to apply the insecticide treated cattle (ITC) method. However, further research is needed to measure the impact of footbaths in areas with higher tsetse densities and wild host densities, that represent interface AAT [36]. The reduction observed in fly catches ascribed to the footbath treatment is mainly attributed to fly kill. It was demonstrated that given the knock-down rates observed against *G* . *p* *. gambiensis* , *G* *. tachinoides* and *G. m. submorsitans* while following the same treatment frequencies applied in this study, a 3% tsetse daily mortality rate could be achieved [21]. The impact at the population level sufficient to suppress isolated tsetse pockets like the ones targeted in Chad [38]. However, a partial repellency is also observed when treating cattle with footbaths using pyrethroids [14], which might increase this killing effect, since unfed flies are more sensitive to deltamethrin by about 50% in comparison to fed flies [39].

Decreasing tsetse populations can contribute to reducing the transmission risk of HAT, even in *T* . *b* *. gambiense* areas. Simple epidemiological models show that a relative density of more than 6 tsetse per human host is necessary for disease transmission in a setting with two hosts, in which the other host is an animal [40]. Recent models show that controlling the disease in the animal reservoir - or preventing its transmission from the animal to the human host - is necessary to eliminate sleeping sickness in humans, even for *T* . *b* *. gambiense* [41]. Regarding *T* . *b* *. rhodesiense* , for which cattle is an important reservoir [42], the impact of footbath treatment is likely to be even more positive by reducing tsetse challenge and minimizing reinfection [18]. In a “single-host” situation where the animal reservoir is not available for tsetse infection (e.g. flies feeding on cattle are killed), the relative density of tsetse flies over human populations must be 24 fold higher for transmission to occur [40]. Footbath treatments present the additional advantage of controlling *A* *. variegatum* ticks before they reach their predilection attachment sites, at least in farming agro-ecosystems of the Sudanese climatic area where cattle spend many hours grazing in natural grasslands [19,43]. The impact on tick infestation is an additional benefit that should encourage adoption of the technology by farmers [38]. Uncontrolled use of footbath treatments might lead to acaricide resistance in tick populations and a possible exacerbation of tick-borne diseases [20]. The risk is low for *A* *. variegatum* because this species, which shows only one generation yearly, temporarily attach to the feet before reaching predilection sites [43]. It is thus subjected to high concentration of acaricide when it first attaches to the host. The situation is more of concern with *Boophilus* species that invade their hosts as larvae through other routes (ears in particular) and for which the restricted application of insecticides might favour prolonged contact with low doses of insecticides. The particular impact of footbath treatment of cattle on ticks and tick-borne diseases was evaluated during this trial, and will be reported separately.

In Burkina Faso, the adoption rate of the insecticide footbath technology by farmers was studied [22]. The quality of technical support provided to farmers had a strong influence. Cattle farmers’ innovation-risk appraisal highlighted individual variations in risk perceptions and benefits, as well as the prominent role of the socio-technical network of cattle farmers [22]. As such, the degree of technical follow-up after the implementation of footbath-treatment control of tsetse populations must be adapted to the farming system and level of autonomy of farmer associations targeted by the development projects. Footbaths will be integrated in the general control strategy of AAT and HAT in Chad (Alfaroukh, pers. com). Considering a fixed cost of nine hundred and eighty euros to build the footbath (and a use duration of 10 years), 72 treatments per year (every week during the dry season and every 3 days during the 4 months rainy season), one initial filling of the footbath and a mean uptake of 200ml per head, the total cost per year is estimated at ~one point five euros per animal for a 100 cattle herd in Chad. A socio-economic survey using questionnaires was organized in March 2010. From 100 farmers surveyed, 78% considered the footbath useful, 7% had no opinion and 4% considered it difficult to use. Moreover, 74% considered it was efficient both against ticks and tsetse, 16% against ticks only, and 10% against tsetse only. Only 60% of them however were ready to invest money to build new footbaths. Regarding organisation, 40% wanted to use a collaborative management scheme within herder associations, 4% wanted to use them on an individual basis, and the remaining believed it should be organized by external actors. Regarding payment frequency, 42% wanted to pay on an individual treatment basis, 38% on a monthly basis, and 20% on a yearly basis. Further sociological studies including a participative approach are needed to evaluate how daily practices of herdsmen can evolve in order to transform this invention into a true innovation tool that is fully integrated into production systems [22].

Restricted applications of insecticides help reduce the amount of insecticide used by up to 90% [16], which might in turn decrease the impact of tsetse control on non-target organisms, particularly in cattle dung [44].

The potential of footbath treatment to control sleeping sickness is an advantageous characteristic, which might favour its adoption by farmers. As mentioned by Wellburn et al. (2006), it would be interesting to combine such vector control methods with the use of trypanocides, that may not achieve an adequate control of trypanosome transmission in humans [45]. This approach of controlling pests of veterinary importance that transmit zoonotic agents is a prime example of the “One Health” concept, where a single vector control technique could mitigate the risk for transmission of AAT in cattle and HAT in humans. As a result, host population vulnerability and disability could be reduced as it has been documented in Kenya’s tsetse habitats or with Gambiense HAT [41,46]. It has also been suggested that footbath treatment could help control malaria when zoophilic mosquito species like *Anopheles* *arabiensis* are the main vectors [47]. This technology might also help prevent the transmission of arboviruses like Rift Valley fever, although the risk of resistance to pyrethroid insecticides like deltamethrin in mosquitoes is high [48].

## Supporting Information

Figure S1 Poisson model of the impact of cattle footbath treatments on tsetse total catches in Chad. (a) comparison of the predicted and observed values, (b) comparison of the random effects related to the intercept of the Poisson model to a normal distribution and (c) standardized differences in the estimation of the fixed effects linked to the individual traps.
