# Supplementary figures and images for: Treating Cattle to Protect People? Impact of Footbath Insecticide Treatment on Tsetse Density in Chad
Source: PLoS One. 2013 Jun 14;8(6):e67580. doi: 10.1371/journal.pone.0067580 (PMC3682971; doi:10.1371/journal.pone.0067580)

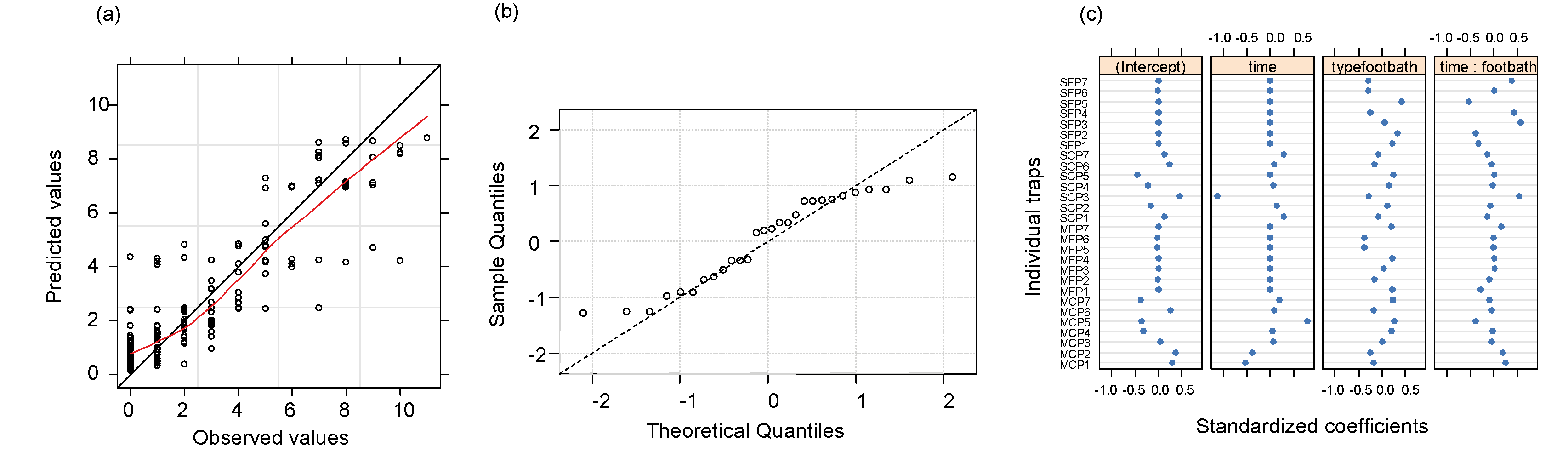

Supplement: Figure S1 — (a) comparison of the predicted and observed values, (b) comparison of the random effects related to the intercept of the Poisson model to a normal distribution and (c) standardized differences in the estimation of the fixed effects linked to the individual traps. [file pone.0067580.s001.tif]
